# A novel TGFbeta/TGILR axis mediates crosstalk between cancer-associated fibroblasts and tumor cells to drive gastric cancer progression

**DOI:** 10.1038/s41419-024-06744-0

**Published:** 2024-05-28

**Authors:** Shanshan Qin, Qiwei Guo, Yue Liu, Xiangang Zhang, Pan Huang, Hedong Yu, Lingyun Xia, Weidong Leng, Dandan Li

**Affiliations:** 1 Department of Stomatology, Taihe Hospital and Hubei Key Laboratory of Embryonic Stem Cell Research, School of Basic Medical Sciences, Hubei University of Medicine, Shiyan, 442000 Hubei China; 2 Laboratory of Tumor biology, Academy of Bio-Medicine Research, Hubei University of Medicine, Shiyan, Hubei P.R. China; 3 Shiyan Key Laboratory of Natural Medicine Nanoformulation Research, Hubei University of Medicine, Shiyan, Hubei 442000 China

**Keywords:** Cancer microenvironment, Gastric cancer, Long non-coding RNAs, Transcriptional regulatory elements

## Abstract

Transforming growth factor beta (TGFβ) signaling plays a critical role in tumorigenesis and metastasis. However, little is known about the biological function of TGFbeta-induced lncRNA in cancer. In this study, we discovered a novel **TG**Fbeta-**i**nduced **l**nc**R**NA, termed **TGILR**, whose function in cancer remains unknown to date. TGILR expression was directly activated by the canonical TGFbeta/SMAD3 signaling axis, and this activation is highly conserved in cancer. Clinical analysis showed that TGILR overexpression showed a significant correlation with lymph node metastasis and poor survival and was an independent prognostic factor in gastric cancer (GC). Depletion of TGILR caused an obvious inhibitory effect on GC cell proliferation, invasion, and epithelial-mesenchymal transition (EMT) in vitro and in vivo. More importantly, we demonstrated that TGFbeta signaling in GC was overactivated due to cancer-associated fibroblast (CAF) infiltration. Mechanistically, increased level of CAF-secreted TGFbeta activates TGFbeta signaling, leading to TGILR overexpression in GC cells. Meanwhile, TGILR overexpression inhibited the microRNA biogenesis of miR-1306 and miR-33a by interacting with TARBP2 and reducing its protein stability, thereby promoting GC progression via TCF4-mediated EMT signaling. In conclusion, CAF infiltration drives GC metastasis and EMT signaling through activating TGFbeta/TGILR axis. Targeted blocking of CAF-derived TGFbeta should be a promising anticancer strategy in GC.

## Highlights

- Increased levels of CAF infiltration led to the overactivation of TGFbeta signaling in GC.
- The induction of TGILR expression by the canonical TGFbeta/SMAD3 signaling axis is highly conserved in cancer.
- The CAF-secreted TGFbeta drives TGILR overexpression in GC cells. TGILR overexpression is clinically associated with lymph node metastasis and poor survival, and plays an oncogenic role in GC.
- TGILR promotes GC progression and EMT by regulating microRNA biogenesis through interacting with TARBP2 and reducing its protein stability.
- CAF drives GC metastasis and EMT signaling through activating TGFbeta/ TGILR axis.

## Introduction

Gastric cancer is one of the cancers with the highest incidence rate and mortality in the world, especially in East Asia [1]. China is a country with a high incidence of stomach cancer, with approximately 400000 new cases and 350,000 deaths each year. Both new cases and deaths account for at least 40% of the world’s GC cases [2]. Metastasis is the main cause of death in GC [3]. About one-third of gastric cancer patients already experience metastasis at the time of treatment, with a 5-year survival rate of less than 10% [4]. The latest molecular typing study indicates that GC patients with epithelial mesenchymal transition (EMT) signatures have the worst prognosis [5]. Thus, it is urgently necessary to understand the molecular mechanism underlying GC metastasis.

Tumor metastasis is a complex biological process involving multiple steps and factors. Tumor cells invade the local area, spread through the bloodstream, and ultimately continue to proliferate and grow in the distal target organ, forming secondary tumors with the same properties as the primary tumor [6]. EMT is considered an important biological process for epithelial-derived malignant tumor cells to acquire migration and invasion capabilities [7, 8]. As a key regulatory cytokine of cellular EMT events, the transforming growth factor beta (TGFbeta) signaling pathway is bound to play essential roles in tumorigenesis and metastasis [9].

TGFbeta is a multifunctional cytokine that has three distinct isoforms, TGFB1, TGFB2, and TGFB3 in mammalian species [10–12]. The molecular mechanism and clinical relevance of TGFbeta signaling in cancer is becoming increasingly clear, paving the way for the development of treatment strategies targeting TGFbeta. TGFbeta promotes tumor progression by regulating the immunosuppressive phenotype [13]. TGFbeta modulates processes such as cell invasion, immune evasion, and microenvironment modification that cancer cells may exploit to their advantage [14, 15]. TGFbeta also promotes tumor progression by regulating downstream proteins and non-coding RNAs [16–19]. These evidences together suggest that TGFbeta signal transduction disorder is one of the dominant factors leading to tumor development.

Recently, mounting evidence has shown that long non-coding RNAs play an essential role in tumor biology through various mechanisms [20–23]. In other words, TGFbeta can theoretically promote tumor progression by inducing the expression of protein-coding genes or non-coding RNAs. However, it is poorly understood which lncRNAs can be induced by TGFbeta as well as their specific biological functions in cancer. In addition, the reason for the over-activation of TGFbeta signaling pathway in tumors is largely unclear.

In this work, we confirmed that the TGFbeta signaling pathway is significant dysregulated in GC due to the increased level of cancer-associated fibroblast infiltration. In addition, we discovered a new **TG**Fbeta-**i**nduced **l**nc**R**NA, termed **TGILR**. LncRNA TGILR (also known as AL590004.3, or RP1 − 140K8.5 or ENSG00000260604) is located at chromosome 6p25.2 and broadly expressed in human tissues. Notably, the induction of the lncRNA TGILR by the canonical TGFbeta/SMAD3 signaling is highly conserved in cancer.

The biological role of lncRNA TGILR remains unknown in cancer. Herein, we reported that lncRNA TGILR directly interacts with the RNA-binding protein TARBP2. TARBP2 is a subunit of RNA-induced silencing complex (RISC) loading complex, which plays a role in regulating microRNA biogenesis [24]. It has been reported that miR-33a and miR-1306 play tumor suppressive roles in GC [25, 26]. In this work, we found that TGFbeta-mediated TGILR overexpression promotes GC progression through regulating microRNA biogenesis of miR-33a and miR-1306 via interacting with TARBP2. Our finding highlights that TGFbeta induced lncRNA TGILR showed a synergistical effect on the induction of EMT signaling in GC.

## Results

### TGFbeta signaling is activated in GC due to the increase of CAF infiltration

There are three isoforms of TGFβ protein, encoded by TGFB1, TGFB2 and TGFB3 genes, respectively. TGFB1 was the most dominant TGFβ isoform in human stomach tissue (Fig. S1). In GC tissues, all three TGFbeta isoforms were significantly dysregulated. TGFB1 and TGFB2 were overexpressed, while TGFB3 was under-expressed in GC (Fig. S2a). Next, we further analyzed their clinical significance in the TCGA (n = 375) and ACRG (GSE62254, n = 300) cohort. The clinical analysis in the two independent GC cohorts both showed that TGFbeta overexpression was significantly associated with poor differentiation, malignant progression, and lymph node metastasis, and predicted a worse prognosis (Figs. S2b-k and S3a-k).

**Fig. 1:**
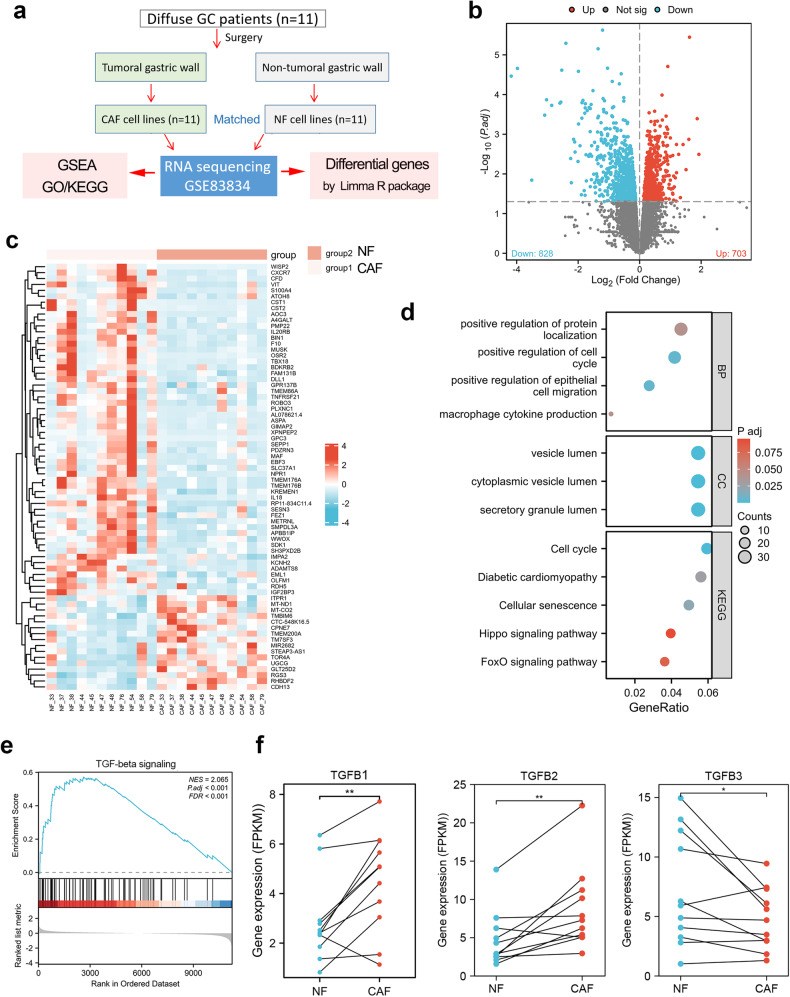
TGFbeta signaling is overactivated in gastric cancer-associated fibroblasts. **a**, **b** The differentially expressed genes (DEGs) between NFs and CAFs were analyzed in GC. **c** The top 100 DEGs between NFs and CAFs were displayed in the heat map. **d** The genes significantly upregulated in CAF were selected to conduct GO/KEGG analysis. **e** The GSEA analysis revealed that TGFbeta signaling was activated in CAF. **f** The expression differences of TGFB1, TGFB2, and TGFB3 between NF and CAF were analyzed. **p < 0.01.

As a secretory cytokine, it is necessary to figure out which cells express these different TGFbeta isoforms. Thus, single-cell analysis was further performed. In both normal stomach and GC tissues, fibroblast is the main and only cell type that expresses all three isoforms of TGFbeta (Fig. S4a–g). On the one hand, we explored the correlation between CAF infiltration and prognosis in GC using the EPIC algorithm (Fig. S5a). The results showed that CAF infiltration was significantly increased and predicted poor prognosis in GC (Fig. S5b–d). On the other hand, we further analyzed the differentially expressed genes (DEG) between NF and CAF in GC. The GSE83834 dataset contains transcriptomic data from 11 pairs of NF and CAF cell lines isolated from cancer and corresponding adjacent tissues, respectively (Fig. 1a). The DEGs between NF and CAF were displayed in the volcano and heat maps (Fig. 1b, c and Table S1). GO/KEGG analysis showed that DEGs were enriched in pathways regarding macrophage cytokine production, cell cycle, vesicle lumen, cellular senescence, and Hippo signaling (Fig. 1d). GSEA analysis showed that the TGFbeta signaling pathways were activated in CAF (Fig. 1e). DEG analysis showed that TGFB1 and TGFB2 were significantly overexpressed, while TGFB3 was significantly downregulated in CAF (Fig. 1f). Taken together, the activation of the TGFbeta signaling pathway in GC is caused by the increased infiltration level of CAF.

### lncRNA TGILR was a novel downstream target gene of TGF-beta signaling

To understand the biological effect of CAF-derived TGFbeta on tumor cells, we conducted RNA sequencing studies in GC cell lines exposed to exogenous recombinant human TGFbeta (rhTGFbeta) active protein (Fig. S6a). GO/KEGG analysis showed that DEGs were enriched in the TGFbeta signaling pathways, suggesting this experiment was successfully conducted (Fig. S6b, c).

**Fig. 2:**
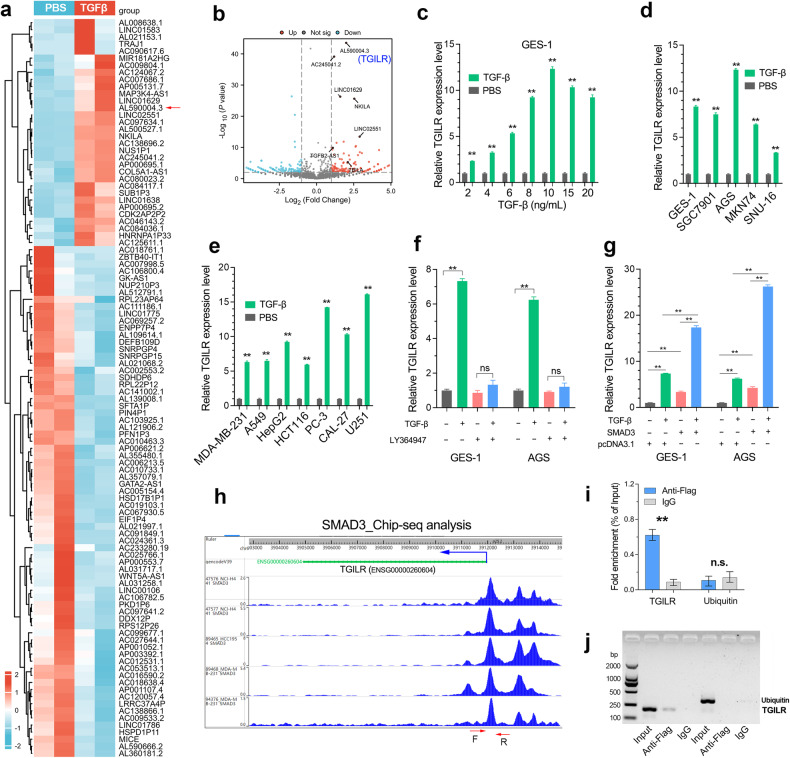
LncRNA TGILR was directly induced by the canonical TGFbeta/SMAD3 axis. **a** Identification of the TGFbeta-regulated lncRNAs in GES-1 cell line by RNA-seq analysis. The differentially expressed lncRNAs (log2FC > 1, p < 0.05) in GES-1 cells exposed to TGFbeta (10 ng/ml) are shown in the heatmap. **b** The log2FC and p values per lncRNA were shown in the volcano plot. TGILR was the most significant lncRNA induced by TGFbeta. **c** TGILR expression can respond to different concentrations of TGFbeta and exhibits a certain concentration dependence in GES-1 cell line. **d**, **e** The phenomenon that TGFbeta activates TGILR expression is widely present in different GC cell lines and different tumor cell lines. **f** The TGFbeta Type I receptor inhibitor LY364947 (5 μM) blocks the induction effect of TGFbeta on TGILR expression in the GES-1 and AGS cell lines. **g** Overexpression of SMAD3 enhanced the induction effect of TGFbeta on TGILR expression in GES-1 and AGS cell lines. **h** Chip-seq analysis by the Cistrome database showed that transcription factor SMAD3 directly binds to TGILR promoter. **i**, **j** A Chip-PCR assay confirmed that transcription factor SMAD3 directly binds on TGILR promoter in the AGS cell line. ****, P < 0.0001; ***, P < 0.001; **, P < 0.01; *, P < 0.05.

Here, we focused on the lncRNAs that are induced by TGFbeta (Fig. 2a). As shown in the heat map, rhTGFbeta treatment caused significant changes (log2FC > 1, p < 0.05) in the expression of over 100 lncRNAs, including the known TGFbeta-induced lncRNA linc02551 [16] and TBILA [27]. Notably, TGILR is the most significantly upregulated lncRNA by rhTGFbeta (Fig. 2b). Subsequent quantitative RT-PCR assays further confirmed that TGILR is induced by rhTGFbeta in a concentration-dependent manner in the GES-1 cell line (Fig. 2c). The induction of TGILR expression by rhTGFbeta protein is widely present in different GC cell lines and human cancer cell lines (Fig. 2d, e), indicating that the regulation of TGILR expression by TGFbeta is highly conserved across human tissues.

According to the current knowledge of TGFbeta signaling, TGFbeta recognizes and binds to its cell membrane receptors, which will activate the phosphorylation of downstream SMAD proteins and promote their entry into the nucleus for transcriptional regulation. LY-364947, a small-molecule compound, is a specific inhibitor for TGFβRI. Blocking TGF signaling with LY-364947 significantly impaired the intense induction of TGILR (Fig. 2f). As transcription factors, SMAD protein can bind to promoters containing SMAD binding elements (SBEs) to regulate gene transcription. Promoter analysis by JASPAR showed that the TGILR promoter region contains several SMAD3 binding sites (CAGACA). Thus, we determined the expression level of TGILR in SMAD overexpression cell lines. The results showed that TGILR expression was greatly upregulated by SMAD3 overexpression. Moreover, there is a synergistic effect between SMAD3 overexpression and rhTGF treatment on the induction of TGILR expression (Fig. 2g). Consistently, chip-seq analysis regarding SMAD3 in lung cancer (NCL-H441) and breast cancer cell lines (HCC1954, MDA-MB-231) together showed that there were 3 SMAD3 binding peaks in the promoter region of TGILR (Fig. 2h). Chip assay in the GC cell line AGS also confirmed that SMAD3 can directly bind to the promoter of TGILR (Fig. 2i, j). Taken together, TGFbeta induced the expression of TGILR through activating SMAD3-mediated transcription.

### TGILR expression is upregulated by CAF-secreted TGFbeta

**Fig. 3:**
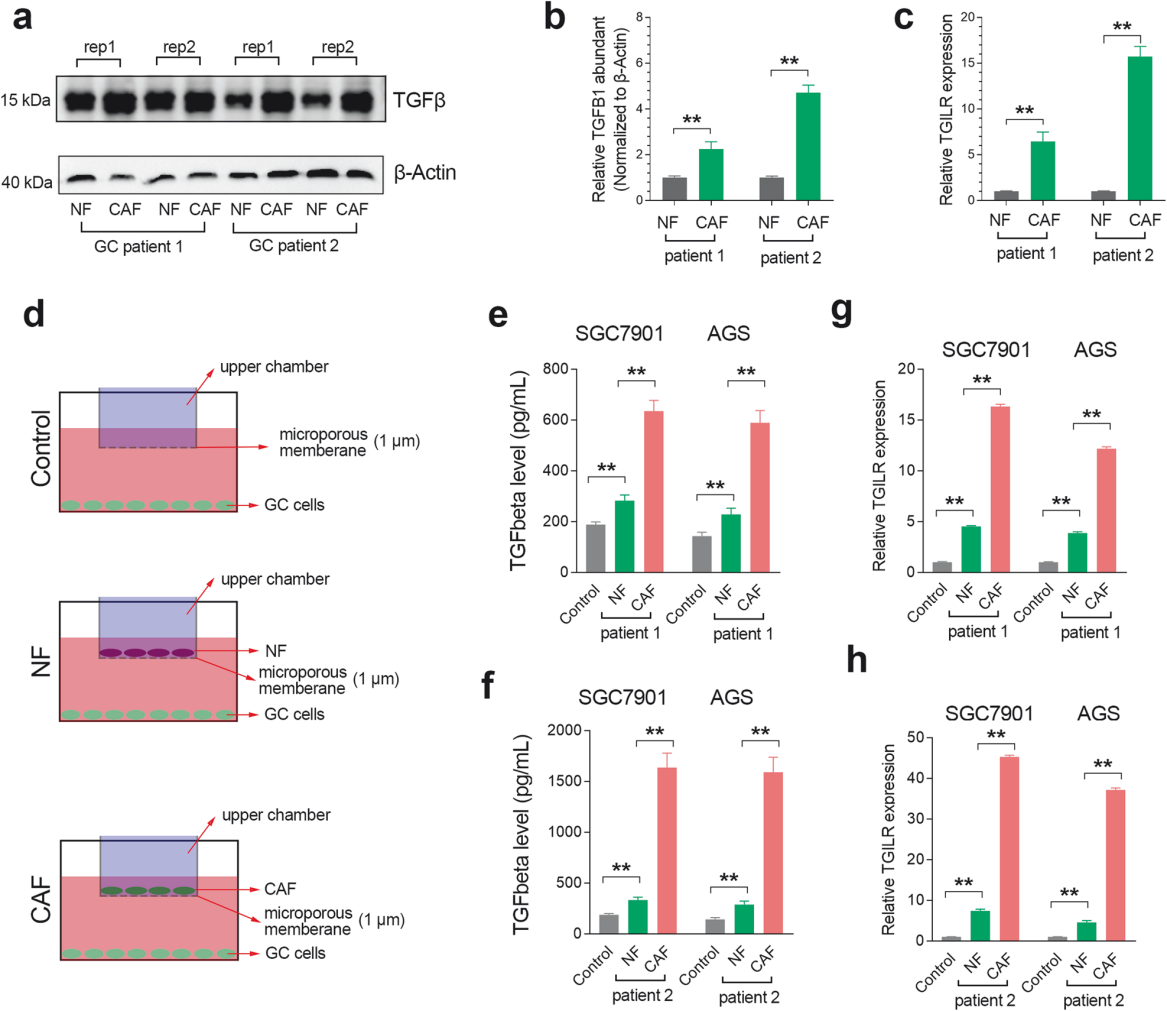
CAF-secreted TGFbeta activates TGILR expression in gastric tumor cells. **a**, **b** Western blotting assays showed that mature TGFbeta protein levels in CAFs were higher than those in NF from the same donor. **c** Quantitative RT-PCR assay showed that TGILR expression was higher in CAF than that in NF from the same donor. **d** Schematic diagram of cell co-culture experiments in different groups. **e**, **f** The ELISA assay showed that the content of TGFbeta in the medium co-cultured with CAF and GC cells was significantly higher than that in the medium co-cultured with NF and GC cells. **g**, **h** Quantitative RT-PCR assays showed that TGILR expression in GC cells co-cultured with CAF was significantly higher than that in GC cells co-cultured with NF. **, P < 0.01.

To investigate the regulatory role of CAF infiltration on TGILR expression, we isolated and obtained NF and corresponding CAF cells from cancer tissue and adjacent tissues of the same donor. The western blotting assay confirmed that the mature TGFbeta protein level in CAF cell lines was significantly higher than that in NF cell lines (Fig. 3a, b). Meanwhile, the expression levels of lncRNA TGILR in CAF cell lines were significantly higher than those in NF cell lines (Fig. 3c). That means the autocrine effect of TGFbeta on CAFs activates the expression level of TGILR.

In addition, we further explored the biological role of CAF-secreted TGFbeta on the expression level of lncRNA TGILR in GC cells using a co-culture system (Fig. 3d). The 1 μm microporous membrane hinders cell penetration but allows secreted proteins to freely pass through. After co-culturing for three days, we detected the content of TGFbeta by ELISA assay and examined the expression level of TGILR in GC cells by quantitative RT-PCR assay. The results showed that the TGFbeta levels secreted by CAFS were higher than those secreted by NF and GC cells (the control group). Besides, qRT-PCR assays verified that the expression level of lncRNA TGILR in CAFs was the highest, followed by the expression level of TGILR in NFs, and the expression level of TGILR was the lowest in the control group of GC cells (Fig. 3e, h).

### TGILR was an independent prognostic biomarker in GC

**Fig. 4:**
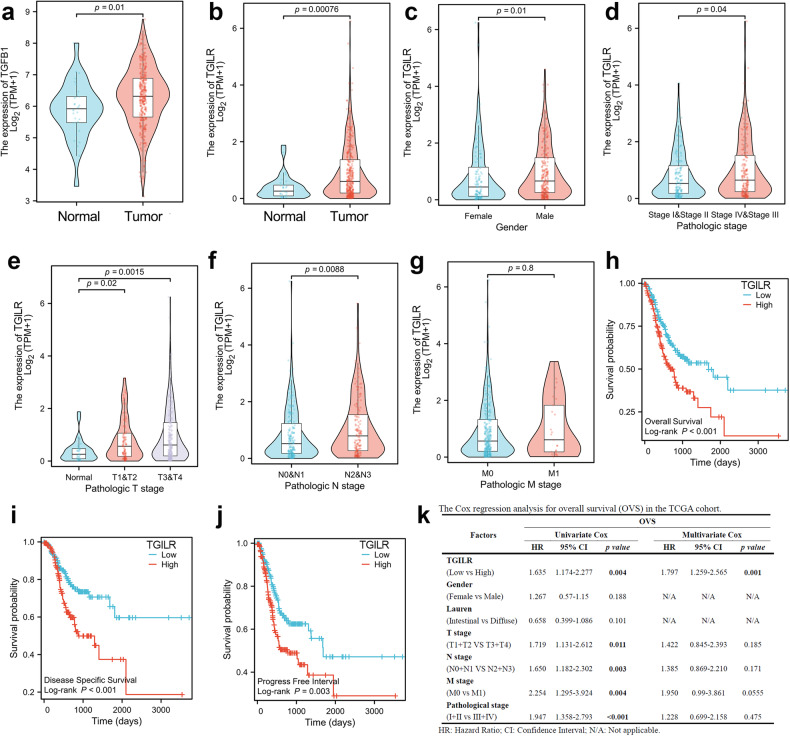
TGILR overexpression was clinically correlated with lymph node metastasis and poor survival. **a**, **b** TGFB1 and TGILR were both significantly upregulated in GC. **c** The correlation of TGILR expression and clinical characteristics was analyzed in GC patients (*n* = 373) from the TCGA cohort. The sex differences in the expression level of TGILR in GC. **d** The expression levels of TGILR in GC patients with different pathologic stages. **e**–**g** The expression differences of TGILR in GC patients with different TNM stages. **h**–**j** GC patients with higher expression of TGILR possessed shorter overall survival (OVS), disease-specific survival (DSS), and progression-free interval (PFI) in GC. **k** Cox regression analysis showed that TGILR was an independent prognostic factor for OVS in GC.

To understand the clinical significance of lncRNA TGILR, we analyzed the clinical correlation between TGILR expression and different clinical characteristics using the TCGA cohort. Expression analysis confirmed that TGFB1 and lncRNA TGILR were significantly overexpressed in GC (Fig. 4a, b). Besides, there was a significant sex differences in the expression level of TGILR (Fig. 4c). As expected, TGILR overexpression showed a significant clinical correlation with pathologic stage (p = 0.04), T stage (p < 0.01) and lymph node metastasis (p < 0.01), but there is no significant correlation with distal metastasis (Fig. 4d–g). Prognostic analysis showed that GC patients with relatively high TGILR expression possessed poor overall survival (p < 0.001), disease-free survival (p < 0.001), and progress-free survival (p < 0.01, Fig. 4h–j). Moreover, Cox regression analysis showed that TGILR expression is an independent prognostic biomarker for the overall survival of GC patients (Fig. 4k).

### TGILR functions oncogenic roles in GC

**Fig. 5:**
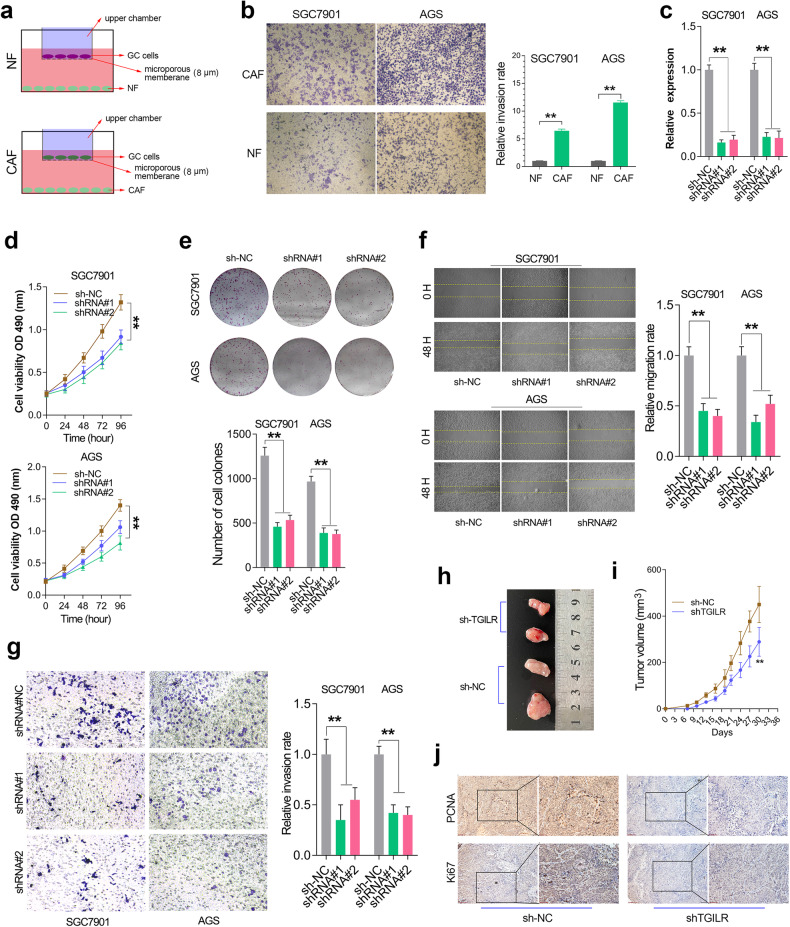
TGILR acts as an oncogene in GC proliferation, migration, and invasion. **a** Schematic diagram of TGILR overexpression on the transwell invasion based on co-culture system. **b** The transwell invasion ability of GC cells co-cultured with CAF was significantly higher than of the GC cells co-cultured with NF. **c** The knockdown efficiency of TGILR in GC cell lines was verified by qRT-PCR assay. **d** The CCK-8 assay showed that knocking down TGILR significantly reduced the growth velocity of GC cell lines. **e**–**g** The colony formation, wound healing, and transwell invasion assays showed that TGILR knockdown significantly inhibited the proliferation, migration, and invasion of GC cell lines. **, P < 0.01. **h** The representative images for the xenograft tumors between TGILR knockdown group and the control group. **i** The growth rate of xenografts formed by TGILR-silenced AGS cells was significantly lower than that of xenografts formed by control AGS cells. **j** The IHC assays in xenografts showed the protein level of cell proliferation biomarkers (PCNA and Ki67) after TGILR knockdown. **, P < 0.01.

Clinical analysis implied that TGILR might act as an oncogene in GC. To verify this speculation, gain-of-function and loss-of -function studies of TGILR were conducted in GC cell lines. Due to the length of TGILR exceeding 7000 bp, directly achieving overexpression by transfection is relatively difficult. We compromised and adopted a CAF-co-culture method to obtain TGILR overexpressing cell lines (Fig. 5a). The subsequent Transwell invasion assay indicated that overexpression of TGILR promoted the invasion ability of GC cells (Fig. 5b). On the other hand, we established GC cell lines with specific knockdown of TGILR using lentiviral transfection (Fig. 5c). To avoid target-off effects, two different shRNA sequences were selected. CCK8 and colony formation assays confirmed that TGILR knockdown significantly suppressed GC cell proliferation (Fig. 5d, e). Wound healing and transwell invasion assays together showed that TGILR knockdown significantly decreased the abilities of GC cell migration and invasion (Fig. 5f, g). In addition, a subcutaneous xenograft tumor mouse model of TGILR_KD was established to determine the biological role of TGILR knockdown on GC cell growth. The results showed that knockdown of TGILR significantly decreased the growth velocity of xenograft tumors (Fig. 5h, i). Moreover, IHC assays in xenograft tumors further showed that TGILR knockdown obviously inhibited the cell proliferation ability of GC cells in vivo (Fig. 5j).

### TGILR interacts directly with TARBP2 and promotes its protein degradation

**Fig. 6:**
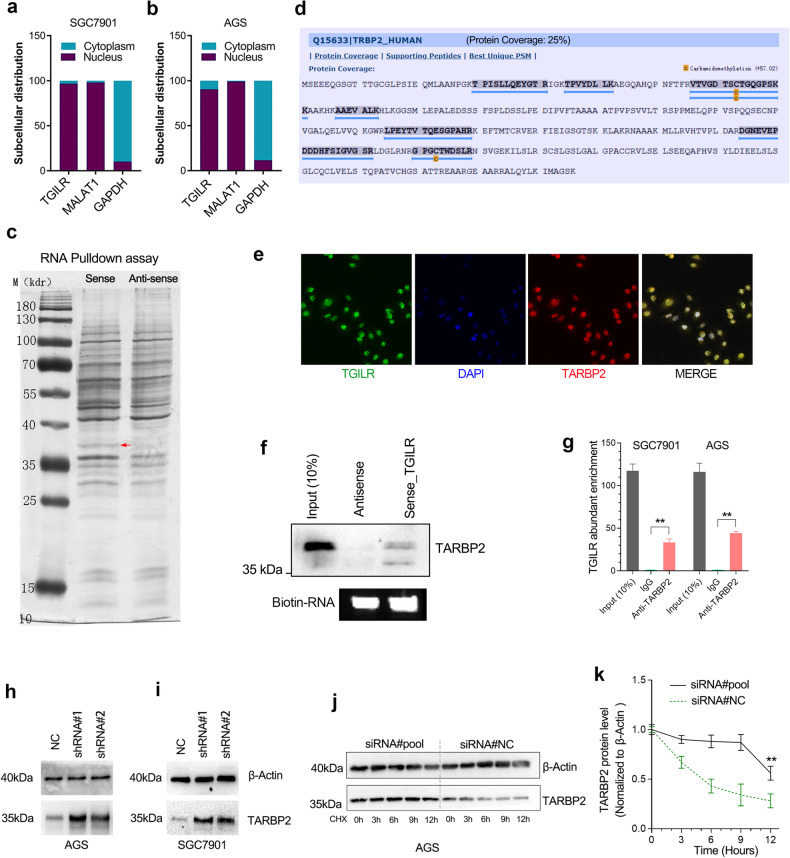
LncRNA TGILR interacts with TARBP2 to regulate its protein stability. **a**, **b** The subcellular location of lncRNA TGILR. **c** RNA pull-down assay was conducted to identify the possible protein interacted with lncRNA TGILR. The sense (S) and anti-sense (AS) of TGILR RNA were biotinylated, refolded, and incubated with AGS cell lysates. The red arrow indicates a possible band specifically bound to TGILR. **d** Mass spectrometry analysis showed that TARBP2 was the most possible protein interacted with TGILR in the designated band. **e** LncRNA TGILR and TARBP2 protein were co-localized in the nucleus of AGS cells. **f** Western blotting assay confirmed the interaction between TGILR and TARBP2. **g**, **h** The RIP-qPCR assay verified the interaction between TGILR and TARBP2. Fold enrichment was determined relative to IgG control. **i** Knockdown of TGILR led to a significant increase in the protein level of TARBP2 in GC cell lines. **j**, **k** The knockdown of TGILR significantly shortened the protein half-life of TARBP2 in AGS cells.

To understand the molecular mechanism of TGILR in regulating GC progression, we first explored the subcellular location of lncRNA TGILR. The nucleo-cytoplasmic RNA separation assay showed that TGILR was a nucleus lncRNA in GC cells (Fig. 6a, b). We speculated that TGILR might function by interacting with RNA-binding proteins (RBPs). Thus, we performed RNA pulldown and mass spectrometry to determine the potential RBPs that can interact with TGILR. A significant differential band located at 35 kDa was removed for mass spectrometry. The mass spectrometry analysis indicated that the protein with the highest abundance in this band was TARBP2 (Fig. 6c, d). Consistently, RNA FISH and immunofluorescence assay confirmed that TARBP2 was a protein co-localized with TGILR (Fig. 6e). Western blotting assays and RNA immunoprecipitation further confirmed that TARBP2 directly interacted with TGILR in GC cell lines (Fig. 6f, g). In addition, we further explored the biological significance of the interaction between TGILR and TARBP2. Western blotting assays showed that the TARBP2 protein level was significantly increased in the TGILR-depleted GC cell lines (Fig. 6h, i). In addition, TGILR knockdown significantly prolonged the half-life time of TARBP2 protein, suggesting that TGILR plays a role in regulating TARBP2 protein stability (Fig. 6j, k).

### TARBP2 exerts tumor suppressive roles in GC by regulating microRNA biogenesis

**Fig. 7:**
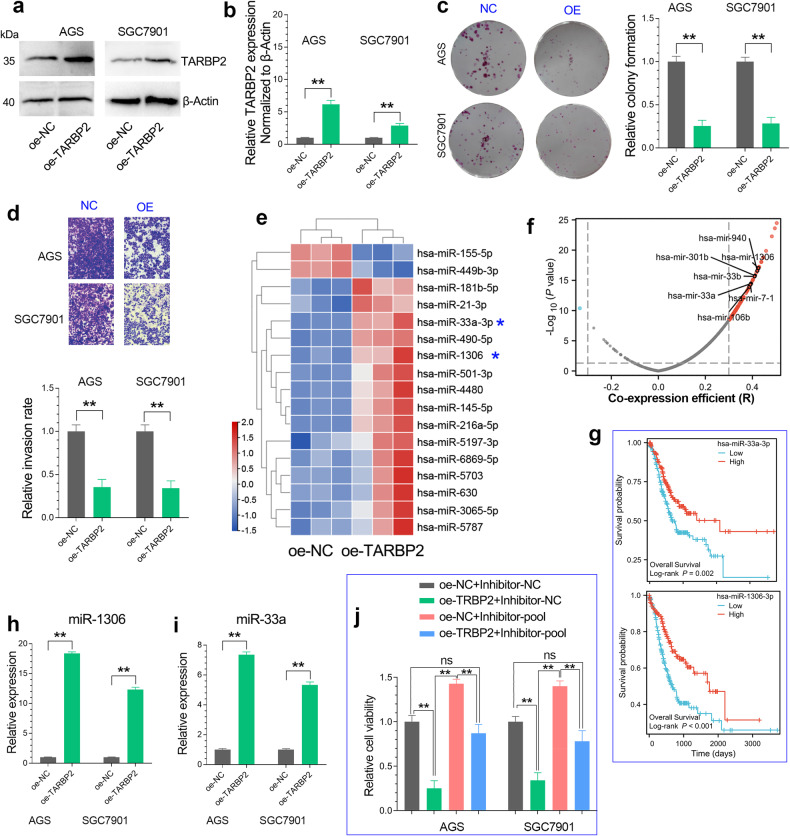
TARBP2 overexpression suppresses GC progression by regulating microRNA biogenesis. **a**, **b** The overexpression efficiency of TARBP2 was examined by western blotting assay. **c**, **d** TARBP2 overexpression significantly inhibits the proliferation and invasion of GC cell lines. **e** The microRNA profiling array was performed to identify the miRNAs regulated by TARBP2 overexpression in the AGS cell line. **f** The expression correlation between TARBP2 and per microRNA in TCGA stomach cancer cohort were shown in the volcano plot. TARBP2 was highly co-expressed with miR-1306 and miR-33a in GC. **g** The overall survival analysis of miR-1306 and miR-33a in the TCGA_STAD cohort. **h**, **i** TARBP2 overexpression significantly upregulated the expression levels of miR-1306 and miR-33a in GC cell lines. **j** The rescue assay confirmed that inhibition of miR-1306 and miR-33a can partially restore the inhibitory effect of TARBP2 overexpression on GC cell proliferation. The inhibitor-poor means the mixture of miR-1306 inhibitor and miR-33a inhibitor.

The biological role of TARBP2 remains largely unclear in GC. To investigate the role of TARBP2 in GC, we established TARBP2 overexpression cell lines by lentivirus transfection (Fig. 7a, b). Colony formation and transwell invasion assays together showed that TARBP2 overexpression inhibited GC cell proliferation and invasion, indicating TARBP2 plays a tumor suppressive role in GC progression (Fig. 7c, d). As an important subunit in the RISC complex, TARBP2 is involved in the biogenesis of microRNAs. Thus, a high-throughput Agilent microRNA profile microarray was performed to identify the differentially expressed microRNAs after TARBP2 overexpression. The heat map showed the top 15 upregulated microRNAs and 2 downregulated miRNAs after TARBP2 overexpression (Fig. 7e). Among them, two microRNAs (miR-1306 and miR-33a-3p) were also highly co-expressed TARBP2 and predicted a favorable prognosis in GC (Fig. 7f, g). To validate the data of microRNA profile microarray, qRT-PCR analysis showed that TARBP2 overexpression significantly increased the expression levels of miR-1306 and miR-33a in GC cell lines (Fig. 7h, i). Rescue assay further confirmed that the inhibition effect of TARBP2 overexpression on GC cell proliferation can be partially restored by miR-1306/33a inhibitors (Fig. 7j). These results suggested that TARBP2 plays a tumor-suppressive role in GC by regulating the microRNA biogenesis of miR-1306 and miR-33a.

### TGILR promotes GC progression by regulating miR-1306 and miR-33a expression

**Fig. 8:**
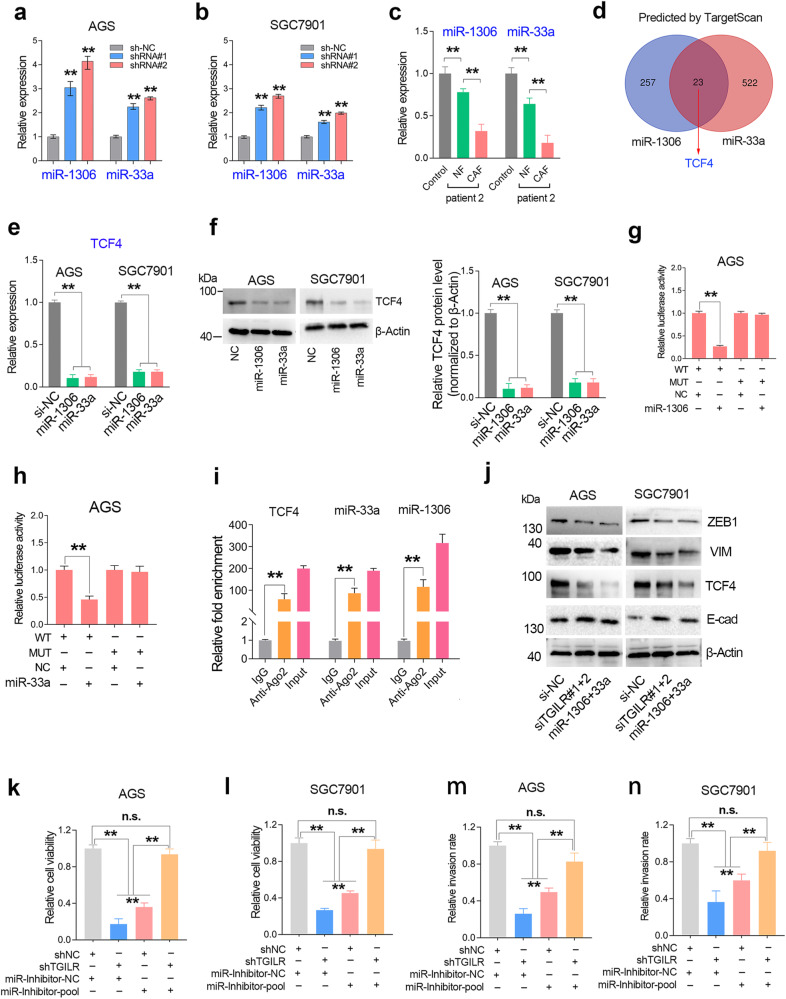
TGILR promotes GC progression and EMT signaling by regulating miR-1306/33a microRNA biogenesis. **a**, **b** Quantitative RT-PCR assays showed that knockdown of TGILR significantly upregulated the expression of miR-1306 and miR-33a in GC cell lines. **c** The expression levels of miR-1306 and miR-33a in GC cells co-cultured with CAF were higher than those in GC cells co-cultured with NF. **d** TargetScan webtool predicted that transcription factor TCF4 was a common target gene of miR-1306 and miR-33a. **e**, **f** Quantitative RT-PCR and Western blotting assays confirmed that TCF4 protein levels were significantly downregulated by miR-1306 and miR-33a in GC cell lines. **g**–**i** Ago2-mediated RIP and luciferase reporter assays showed that TCF4 was directly targeted by miR-1306 and miR-33a in GC. **j** Both TGILR knockdown and miR-1306/33a overexpression significantly inhibit TCF4 expression and the EMT signaling pathway in GC cell lines. The siTGILR#1 + 2 means the mixture of two different siRNAs targeting TGILR. The miR-1306 + 33a means a mimics mixture of miR-1306 and miR-33a. **k**, **l** The rescue proliferation assay showed that inhibiting the expression of miR-1306/33a in stable TGILR-silenced GC cell lines can salvage the inhibitory effect of TGILR knockdown on GC cell proliferation. **m**, **n** The rescue invasion assay showed that inhibiting the expression of miR-1306/33a in stable TGILR-silenced GC cell lines can partially restore the inhibitory effect of TGILR knockdown on GC cell invasion.

Next, we further examined the expression level of miR-1306 and miR-33a in the TGILR-depleted GC cell lines. The results showed that TGILR knockdown increased the expression level of miR-1306 and miR-33a in GC cell lines (Fig. 8a, b). Besides, cell co-culture assays confirmed that CAF-mediated TGILR overexpression significantly reduced the expression levels of miR-1306 and miR-33a in GC cells (Fig. 8c). Bioinformatics analysis by TargetScan web tool showed that an EMT-related transcription factor TCF4 was a common target gene of miR-1306 and miR-33a (Fig. 8d). Quantitative RT-PCR and western blotting assays confirmed that TCF4 was targeted by the mimics of miR-1306 and miR-33a (Fig. 8e, f). The Ago2-RIP assay and luciferase assay together confirmed that TCF4 was directly co-targeted by miR-1306 and miR-33a in GC (Fig. 8g, i). Western blotting assays showed that TGILR knockdown and miR-1306/33a overexpression have similar effects on TCF4-mediated EMT signaling (Fig. 8j). Consistently, the rescue assays further confirmed that TGILR knockdown inhibited GC proliferation and invasion in a miR-1306/33a-dependent manner (Fig. 8k–n).

**Fig. 9:**
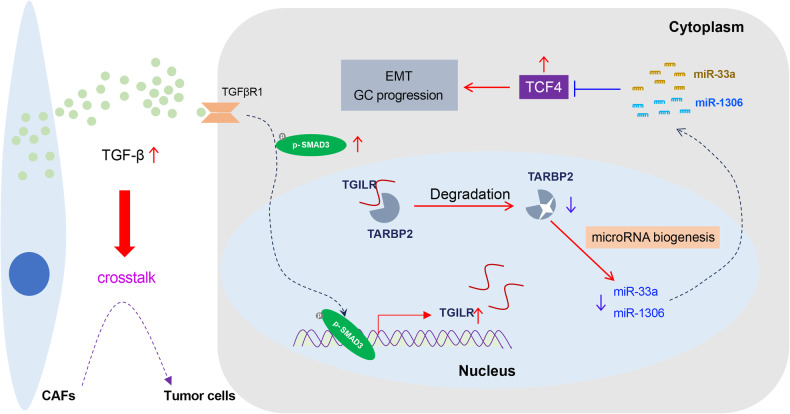
The working model for the cross-talking between cancer-associated fibroblasts and gastric cancer cells mediated by the TGFbeta/TGILR axis.

In conclusion, we proposed a working model of the carcinogenic effect of TGILR (Fig. 9). Briefly, increased CAF infiltration level predicted poor prognosis in GC. CAF infiltration led to the abnormal activation of TGFbeta signaling in GC. More importantly, TGILR was a novel lncRNA induced by TGFbeta signaling. TGILR was transcriptionally regulated by SMAD3. TGILR overexpression promoting GC progression by interacting with TARBP2 and promoted its protein degradation. TARBP2 plays a role in microRNA biogenesis of miR-1306 and miR-33a, which exert tumor suppressive roles by co-targeting TCF4 in GC. Our finding highlighted that lncRNA TGILR promoted GC progression by regulating microRNA biogenesis via TARBP2 degradation.

## Discussion

Tumor microenvironment is a complex structure composed of tumor cells, stromal cells, cancer associated fibroblasts (CAFs), endothelial cells and immune cells. Secretory proteins produced by these cells can mediates the cell–cell communication via autocrine or paracrine mechanisms. The secretory proteins derived from CAFs together constitute the secretome of CAFs, which play a critical role of in tumorigenesis and metastasis by regulating cell communication between CAFs and other cells [28]. For example, we recently reported that CAF-derived FGF2 mediated the crosstalk between CAFs and GC cells through FGFR1-dependent manner [29]. Similarly, we additionally found that CAF-secreted IGFBP7 protein promotes GC progression via regulating macrophage polarization [4]. Here, we focus on the biological functions of TGFbeta, a well-known CAF secretion protein.

Transforming growth factor beta (TGF-β) is a crucial secreted cytokine that has gained increasing concern in recent years for human diseases [30]. TGFbeta family members have multiple impacts on the regulation of cell fate during embryonic development and tissue homeostasis through canonical and/or noncanonical signaling pathways [31]. In noncanonical signaling, TGFbeta members can conduct signal transduction without relying on SMADs [32]. On the contrary, the TGFbeta/SMAD axis is the canonical and main signaling pathway through binding to TGFbeta receptors in endocrine, paracrine, and autocrine settings [33]. Li et al. have reported that dysregulation of the TGFβ/SMAD4 signaling plays an essential role in the metastatic properties and cell fate decisions of pancreatic cancer cells [34]. Fang and colleagues emphasized that abnormal activation of SMAD3 might be a critical node of the canonical TGFbeta signaling in granulosa cell tumor development [35].

In this study, we highlighted that the abnormal activation of the TGFbeta signaling pathway in GC is due to the increased of CAF infiltration. Single-cell analysis showed that fibroblast is the main cell type expressing all three TGFbeta isoforms. In this case, the increase in CAF infiltration inevitably leads to the upregulation of TGFbeta in GC. Consistently, Ishimoto et al. also reported that the TGFbeta signaling pathway is significantly upregulated in gastric CAF [36]. In addition, we identified TGILR (AL590004.3 or ENSG00000260604) as a novel TGFbeta-induced lncRNA in GC. TGFbeta activates TGILR expression through canonical pathways in a SMAD3-dependent manner. More importantly, the induction of TGILR expression by TGFbeta signaling is highly conserved in different human cancer cell lines, indicating that TGILR may play an important role in cancer.

The biological function of TGILR in cancer has not been reported yet. Herein, we show that TGILR overexpression is clinically associated with malignant progression, lymph node metastasis, and is an independent prognostic factor for survival in GC patients. Besides, loss-of-function studies showed that TGILR acts as an oncogene in GC by promoting cell proliferation and metastasis in vivo and in vitro. Additionally, we identified that the TGFbeta/TGILR axis was involved in the crosstalk between CAF and gastric cancer cells.

LncRNA can act as scaffolds, decoys, guides, and signals to participate in diverse processes such as epigenetic modification, transcriptional regulation, protein translation, RNA/protein stability, and miRNA sponge, thereby exerting biological roles in diverse diseases, especially cancer [37]. In this work, we identified a strong physical combination between lncRNA TGILR and the RNA-binding protein TARBP2. And this RNA-protein interaction plays a role in repressing the protein stability of TARBP2. As a subunit in RISC complex, TARBP2 plays significant roles in many biological and pathological conditions, including viral expression of HIV-1, microsatellite instability, cancer stem cell properties, and tumor progression [38]. Increasing studies have reported that TARBP2 is involved in the process of microRNA biogenesis [24, 39]. In this study, we showed that TARBP2 overexpression inhibited GC progression by regulating the expression of two tumor suppressive microRNAs (miR-1306 and miR-33a) [25, 26]. Additionally, we further confirmed that the EMT-related transcription factor TCF4 was a putative target gene of miR-1306 and miR-33a [40]. TGILR knockdown and miR-1306/33a overexpression have a similar inhibitory effect on EMT signaling. These results suggested that the TGFbeta-induced lncRNA TGILR synergistically plays a role in the induction of EMT signaling by TGFbeta. Rescue assay further confirmed that TGILR promotes GC progression by regulating the expression of miR-1306/33a through interaction with TARBP2.

In summary, increased CAF infiltration leads to overactivation of TGF-beta signaling in GC. The activation of the TGF signaling pathway can cause significant changes in the expression of many genes, including a novel lncRNA TGILR. TGILR plays a oncogenic role in GC by enhancing the microRNA biogenesis of miR-1306/33a and maintaining TCF4 overexpression through interaction with TARBP2. Our finding highlights that CAF infiltration promotes GC progression by TGFbeta/TGILR axis.

## Methods

### Clinical analysis and immune infiltration

This study was approved by the Human Research Ethics Committee of Hubei University of Medicine. The global gene expression profiling of GC samples from TCGA_STAD and GSE62254 cohorts was downloaded as we previously described [41, 42]. The clinical significances of TGILR and TGFbeta isoforms were determined by the correlation between gene expression levels and clinical characteristics of GC patients. For immune infiltration analysis, the immune infiltration level of CAF between stomach cancer (TCGA) and normal stomach tissues (GTEx) was analyzed using the GEPIA 2021 web tool. Based on the inferred cell proportions in each bulk RNA sample, GEPIA2021 web tool was used to conducted proportion analysis, correlation and survival analysis. The EPIC method was selected since it can estimate the immune infiltration of macrophages and CAFs.

### Single-cell analysis

A published single-cell RNA-seq (scRNA-seq, GSE167297) dataset of diffuse GC samples was downloaded from the NCBI GEO repository [43]. The single-cell analysis using R package Seurat (version 4.1.0) was performed as we previously described [44]. Briefly, once the quality control steps were completed, data normalization and scaling were conducted using the Seurat package. The top 3000 variable genes for all samples were used for principal component analysis (PCA). The cluster of different cell types was performed by the corresponding classical biomarkers. The scRNA-seq analysis in the normal human stomach tissues was directly screenshotted from the human protein atlas (HPA) web tool.

### DEGs between NF and CAF

The GSE83834 database contained the transcriptome data of 11 paired NF and CAF cell lines. To screen for significant differentially expressed genes (DEGs) between normal fibroblast (NF) and CAF, we downloaded the global gene expression data (normalized FPKM data) of GSE83834 from the Gene Expression Omnibus (GEO) repository. Then, the Limma R package was performed to calculate the log2FC and P values of per gene [29].

### Cell culture and establishment of cell lines

The GC cell lines and the normal gastric cell line GES-1 used in this study were purchased from the Shanghai Cell Bank of the Chinese Academy of Sciences. For cell culture, the cell lines used in this study were cultured in DMEM medium containing 10% fetal bovine serum (FBS) at 37 ^o^C in 5% CO_2_. For stable knockdown of TGILR, the lentiviruses of shTGILR were purchased from Genepharma. Lentiviral transfection was performed according to the manufacturer’s instructions. For transient transfection experiments, the siRNA information used in this study was listed in Table S2. At the indicated time points, the cells were harvested for mRNA and protein analysis as well as for other assays. For TGFbeta treatment, cells were seeded into 6-well plates and grown overnight. The next day, when the cell plating density reached 50–70%, cells were treated with 10 ng/ml recombinant human TGFbeta protein (HZ-1011, Proteintech, Wuhan, China).

### RNA sequencing

The GES-1 cells were harvested to extract total RNA at 48 h after TGFbeta or PBS treatment. The library construction and sequencing of RNA samples were conducted in Shanghai Lifegenes Technology Company using Illumina NovaSeq 6000. The RNA-seq data was uploaded to the GEO section of the NCBI web server. The GEO accession number was GSE245557.

### MicroRNA array

The expression profile of miRNAs after TARBP2 overexpression was detected by a high-throughput microRNA profiling array (miRNA 8X60K microarray, Agilent), which was purchased from Superchip (Shanghai, China). Briefly, after overexpression of TARBP2 in GC cells, the total RNA of each sample was extracted. After passing the RNA quality control, the total RNA samples were shipped on dry ice to Superchip Company (Shanghai, China) for microRNA array studies. After normalization by the coefficient of variation (CV value) of each probe, the relative expression level of each microRNA was calculated. The foldchange value was calculated to estimate between-group differences.

### Quantitative RT-PCR assay

The cellular total RNA samples were extracted using Trizol reagent (Invitrogen, USA) according to the manufacturer’s instructions. The cDNA samples were obtained using the PrimeScript^TM^ RT reagent Kit (Perfect Real Time, Takara). The quantitative RT-PCR assay was conducted using Bio-Rad CFX Manager 3.1 real-time PCR system. The specific primers used in this study were listed in Table S3. The 2 ^–∆∆Ct^ method was used to determine relative gene expression quantification.

### Mouse xenograft model

The animal experiments were was approved by the Committee on Animals of the Hubei University of Medicine. Four-week-old female BALB/c nude mice were purchased from the Laboratory Animal Center of Hubei University of Medicine. SGC7901 cells in shNC and shTGILR groups were injected into the subcutaneous tissue of female BALB/c nude mice. After 28 days, all the mice were killed, and the tumors were collected for weighing and volume measurement. The tumor volume was calculated using the following formula: volume = length × (width)^2^/2.

### Cell co-culture

CAF/NFs were acquired from two diffuse stomach cancer patients who underwent a surgery at the Taihe Hospital, and the use of specimens was approved by the Institutional Review Board of Hubei university of medicine. The CAFs isolated from cancer tissue and NFs isolated from adjacent tissues are from the same donor. Briefly, tissues were cut into small pieces and digested with collagenase type I (1 mg/mL; Sigma) and hyaluronidase (125 units/mL; Sigma) for 6 h in DMEM without FBS at 37 °C. After filtering the undigested tissues, the stromal fraction was centrifuged at 1000 rpm for 5 min.

### Subcellular location

Briefly, GC cell lines were seeded and fixed with 4% paraformaldehyde. The next day, when the cell plating density reached 50–70%, GC cells were treated with 0.5% Triton, followed by pre-hybridization. Overnight hybridization was performed with a 10 mM probe concentration. The RNA FISH kit was purchased from RiboBio (Guangzhou, China). The experiment was performed according to the manufacturer’s instructions. The 5’FAM-TGILR probes were designed and synthesized by Sangon Biotech (Shanghai). Images were taken with a confocal microscope (Zeiss).

### Chromatin immunoprecipitation and western blot assay

Chromatin immunoprecipitation (CHIP) and western blotting assays were performed as we previously described [22]. For CHIP-PCR analysis, the GC cells were collected and fixed with 1% formaldehyde. After DNA shearing, protein and DNA immunoprecipitation, cross-linked DNA reversal, and DNA purification, the final immunoprecipitated DNA fragments were detected by quantitative PCR and gel electrophoresis assays. For western blotting assay, the primary antibody information including product number and dilution ratio were listed in Table S4.

### RNA pulldown and RNA immunoprecipitation assay

The RNA pulldown and RNA immunoprecipitation (RIP) assays were performed as previously described [21, 22]. For RNA pulldown, the biotin-labeled sense and antisense TGILR RNA samples were incubated with cell lysates overnight at 4 °C with agitation. After washing, the pulldown proteins were prepared for SDS-PAGE, western blot assay and mass spectrometry (MS) analysis. For RIP assay, after crosslinking with 0.5% formaldehyde for 10 min at room temperature, cells were harvested in RIP lysis buffer. Supernatants were incubated with the anti-TARBP2 antibody (Proteintech, China) or IgG overnight at 4 °C. Then, the immunocomplexes of proteins and RNAs were de-crosslinked at 95 °C for 15 min. The immunoprecipitated RNAs were then purified for qRT-PCR analysis.

### Statistical analysis

For gene expression analysis in different subtypes of GC, the P values were estimated using the Mann-Whitney nonparametric test. Survival curves were calculated using the Kaplan-Meier method, and differences between the curves were analyzed using the log-rank test. All the rest of the experiments used unpaired *t*-test or one-way ANOVA test. For all experiments, a minimum of triplicates per group and repetition at least three times were applied to achieve reproducibility. All tests with p values less than 0.05 were considered statistically significant.

### Supplementary information

Supplementary Table S1

Supplementary Figures and Tables

Original WB images

## Data Availability

The data that support the findings of this study are available from the corresponding author upon reasonable request.
