# Supplementary material for: A novel TGFbeta/TGILR axis mediates crosstalk between cancer-associated fibroblasts and tumor cells to drive gastric cancer progression
Source: Cell Death Dis. 2024 May 28;15(5):368. doi: 10.1038/s41419-024-06744-0 (PMC11133402; doi:10.1038/s41419-024-06744-0)
Supplement: Supplementary file 2 — Supplementary Figures and Tables [file 41419_2024_6744_MOESM2_ESM.pdf]

## Supplementary Tables

**Table S1.** The log2FC and p value of the DEGs after RNA-seq analysis of the GSE83834 dateset.

**Table S2.** The microRNA mimics and inhibitors used in this study.

| Gene Name | Sequence (5→`3`) | |
|---|---|---|
| siRNA | | |
| Mimics-NC | UUCUCCGAACGUGUCACGUTT | ACGUGACACGUUCGGAGAATT |
| miR-1306_mimics | CCACCUCCCCUGCAAACGUCCATT | UGGACGUUUGCAGGGGAGGUGGTT |
| miR-33a_mimics | GUGCAUUGUAGUUGCAUUGCATT | UGCAAUGCAACUACAAUGCACTT |
| Inhibitor_NC | CAGUACUUUUGUGUAGUACAA | N/A |
| miR-1306_inhibitor | UGGACGUUUGCAGGGGAGGUGG | N/A |
| miR-33a_inhibitor | UGCAAUGCAACUACAAUGCAC | N/A |
| Inhibitor_pool | UGGACGUUUGCAGGGGAGGUGG | UGCAAUGCAACUACAAUGCAC |
| miRNA_RT | GTCGTATCCAGTGCGTGTCGTGGAGTCGGCAATTGCACTGGATACGACCGGCCTG | |

**Table S3.** Primers used in this study.

| Gene Name | Sequence (5→`3`) | |
|---|---|---|
| | Forward primer | Reverse primer |
| TGILR_qPCR | AGGGAGCGCTAACAGTGAAA | GCAGGGGCGTTATAAATGAA |
| MALAT1-qPCR | AAAGCAAGGTCTCCCCACAAG | GGTCTGTGCTAGATCAAAAGGCA |
| GAPDH-qPCR | TCACCAGGGCTGCTTTTA | AAGGTCATCCCTGAGCTGAA |
| TARBP2-qPCR | GGAGCCTGATGATGACCACT | CTCAGGCTCAGCTCCTCAAT |
| ACTIN_qPCR | ATCGTCCACCGCAAATGCTTCTA | AGCCATGCCAATCTCATCTTGTT |
| Chip-TGILR | CAGCTCCTGTTTCTCCTTGG | CAAGGAGGGGGAAAGAAAAC |
| Chip-Ubiquitin | TGGGTCCGATTATTGAATGG | AGCTGGGTGTCCAGGTTAAA |
| miRNA qPCR | Forward primer | Reverse primer |
| U6 | CTCGCTTCGGCAGCACA | AACGCTTCACGAATTTGCGT |
| miR-1306 | GGGGCCACCUCCCCUGCAAA | CAGTGCGTGTCGTGGAGT |
| miR-33a | GGGGUGCAUUGUAGUUGCA | CAGTGCGTGTCGTGGAGT |

**Table S4.** The information of antibodies used in this study.

| Protein Name | Brand | Catalog NO. | Dilution ratio |
|---|---|---|---|
| GAPDH | Proteintech | 60004-1-Ig | 1:5000 |
| β-Actin | ABclonal | AC026 | 1:10000 |
| CDH1/E-cad | ABclonal | A3044 | 1:4000 |
| TCF4 | ABclonal | A15000 | 1:1000 |
| TGFbeta | Proteintech | 21898-1-AP | 1:2000 |
| TARBP2 | Proteintech | 15753-1-AP | 1:1000 |
| ZEB1 | Proteintech | 21544-1-AP | 1:1000 |

## Supplementary Figures

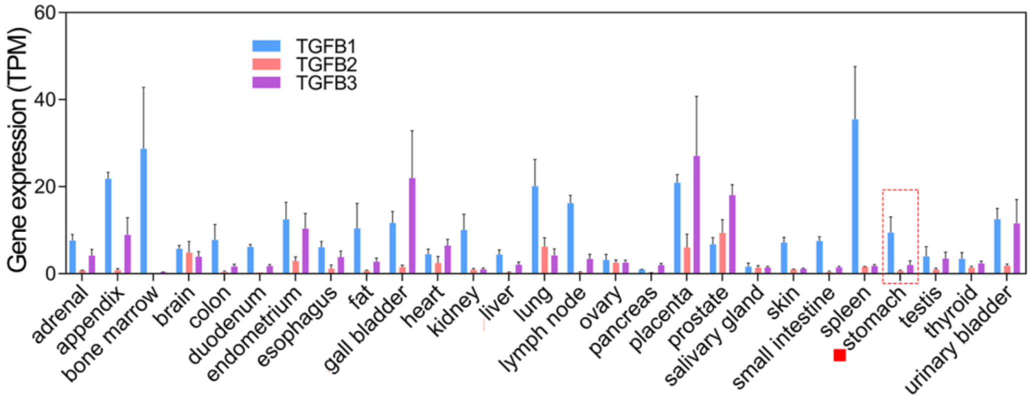


**Figure S1.** The expression pattern of TGFbeta family members in different human tissues. TGFB1 is the dominant TGFbeta isoforms in human stomach tissue.

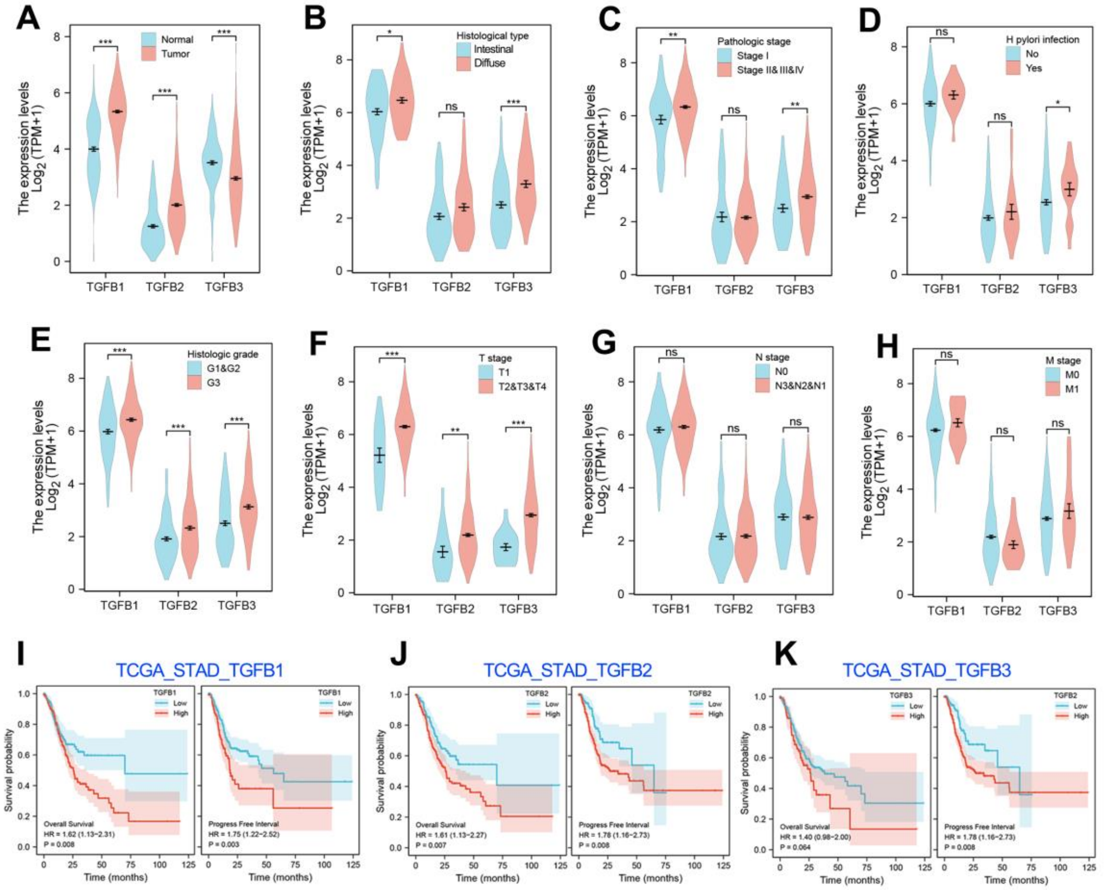


**Figure S2.** The prognostic significances of TGF-β superfamily were analyzed in the GC cohort from TCGA. **(A)** TGFB1 and TGFB2 was overexpressed in GC, TGFB2 was under-expressed in GC. **(B)** The expression difference of TGF-β superfamily in intestinal and diffuse GC. **(C)** The expression level of TGF-β superfamily in different pathologic stages. **(D)** Overexpression of TGF-β superfamily was associated with poorly differentiated GC. **(E)** The expression difference of TGF-β superfamily in GC patients with/without H. pylori infection. **(F-H)** The expression level of TGF-β superfamily in GC tissues with different TNM stages. **(I-K)** The overall-survival and progression-free survival analysis of TGF-β superfamily in GC. **, $P < 0.01$.

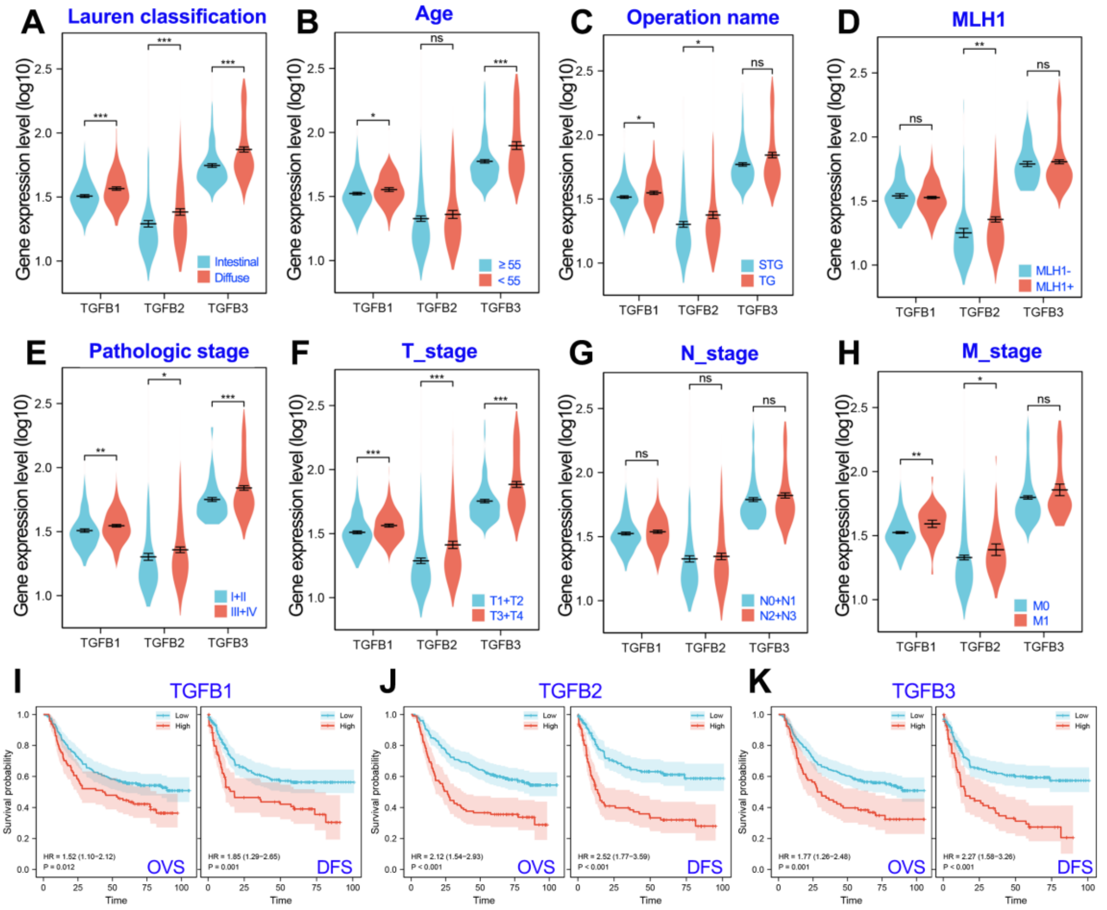


**Figure S3.** The clinical value of TGF-β superfamily was analyzed in the GC cohort from GSE62254. **(A)** The expression difference of TGF-β superfamily in intestinal and diffuse GC. **(B)** The expression difference of TGF-β superfamily between gastric cancer patients over 55 years old and patients under 55 years old. **(C)** The expression difference of TGF-β superfamily between gastric cancer patients with pylorus-sparing radical gastrectomy (STG) operation or total gastrectomy (TG) operation. **(D)** The expression difference of TGF-β superfamily between gastric cancer patients with/without MLH1 expression. **(E)** The expression difference of TGF-β superfamily in GC patients with different pathologic stages. **(F-H)** The expression level of TGF-β superfamily in GC tissues with different TNM stages. **(I-K)** The overall-survival and progression-free survival analysis of TGF-β superfamily in GC. **, P < 0.01.

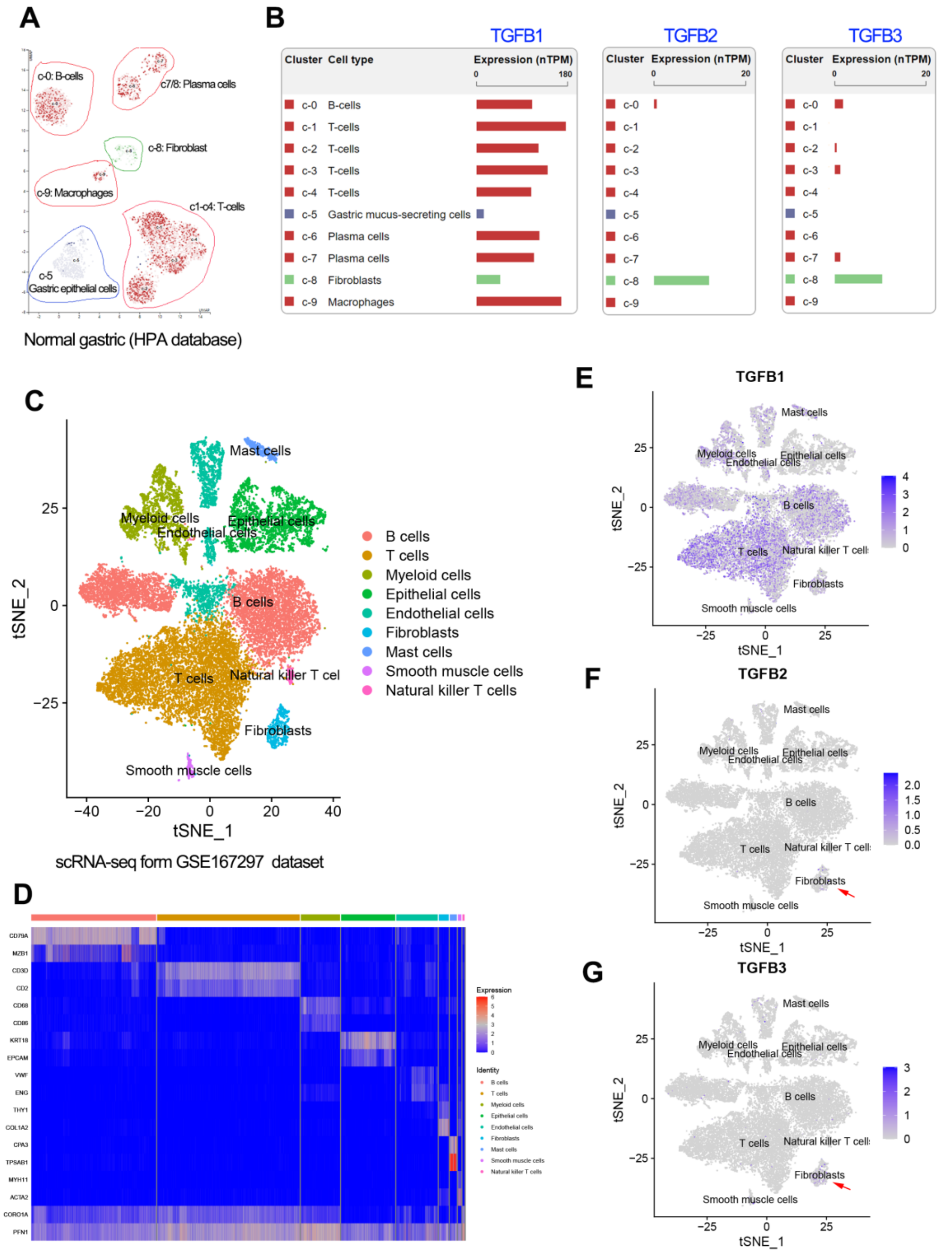


**Figure S4. Single-cell analysis reveals the cell-type specificity of different TGF-β isoforms**. **(A-B)** Single-cell analysis in normal gastric tissues reveals the cell-type specificity of TGFB1, TGFB2 and TGFB3. The related data was obtained from the HPA dataset. **(C)** UMAP plots of the scRNA-seq datasets in GC tissues displayed by cell

type. **(D)** The biomarkers used to distinguish different cell types in GC. **(E-G)** The expression of TGFB1, TGFB2 and TGFB3 in each cell type.

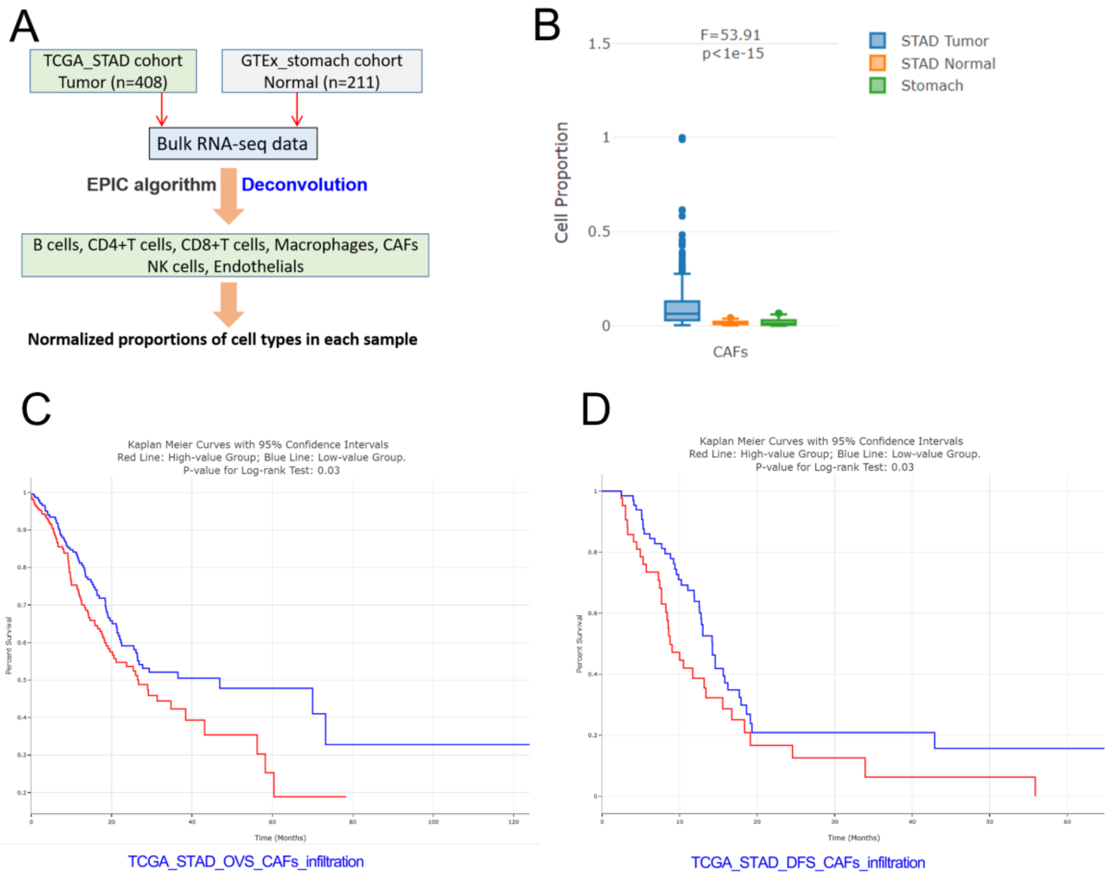


**Figure S5.** Increased CAF infiltration level predicted poor prognosis in GC. **(A)** Schematic diagram of deconvolution-based analysis in normal stomach (GTEx cohort) and GC tissues (TCGA_STAD cohort). **(B)** The proportion analysis of CAF infiltration using EPIC method. **(C, D)** The overall-survival (OVS) and disease-free survival (DFS) analysis of CAF infiltration was performed in GC. **, $P < 0.01$.

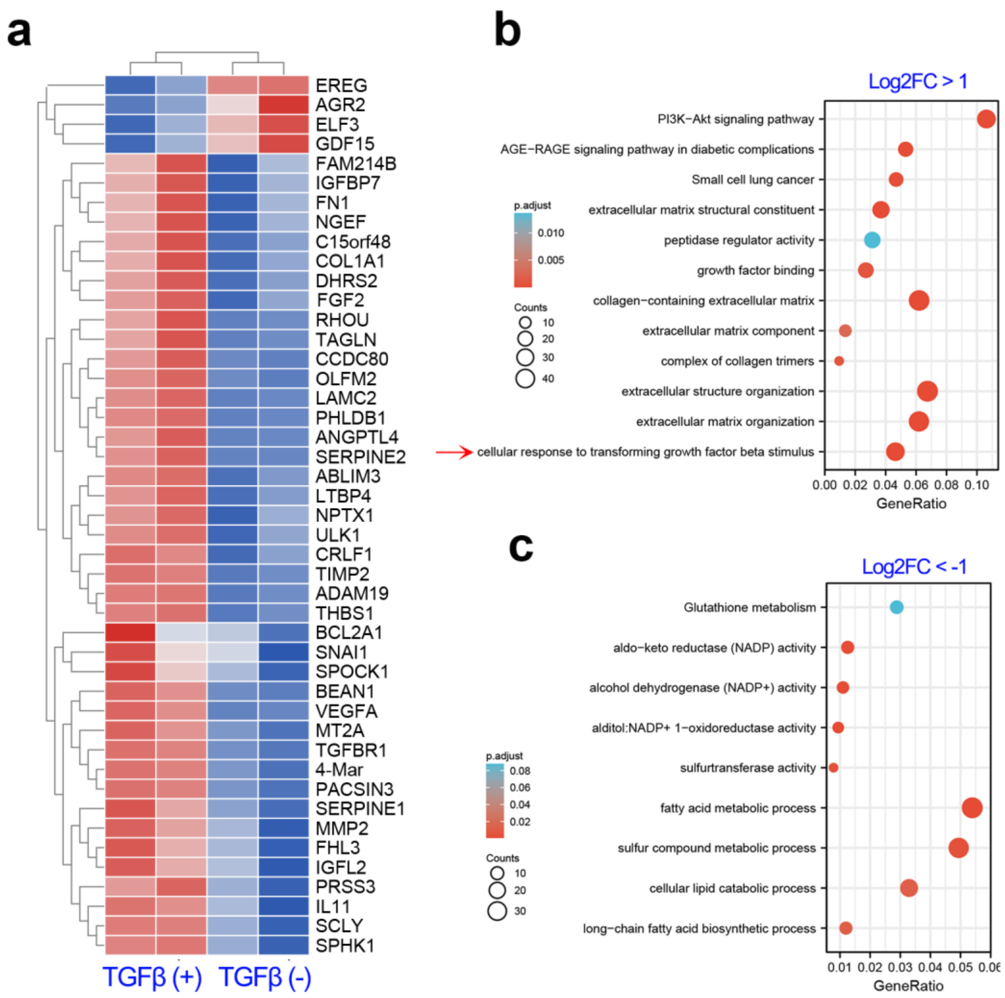


**Figure S6.** The differential expressed protein-coding genes and signaling pathways in GES-1 cell line exposed to TGFbeta. **(A)** The most significant differential expressed protein-coding genes (log2FC>2, p<0.05) in GES-1 cell line exposed to TGFbeta (10 ng/ml). **(B, C)** The genes significantly upregulated or downregulated by TGFβ were selected to conducted GO/KEGG analysis.
