# Supplementary figures and images for: A novel TGFbeta/TGILR axis mediates crosstalk between cancer-associated fibroblasts and tumor cells to drive gastric cancer progression
Source: Cell Death Dis. 2024 May 28;15(5):368. doi: 10.1038/s41419-024-06744-0 (PMC11133402; doi:10.1038/s41419-024-06744-0)

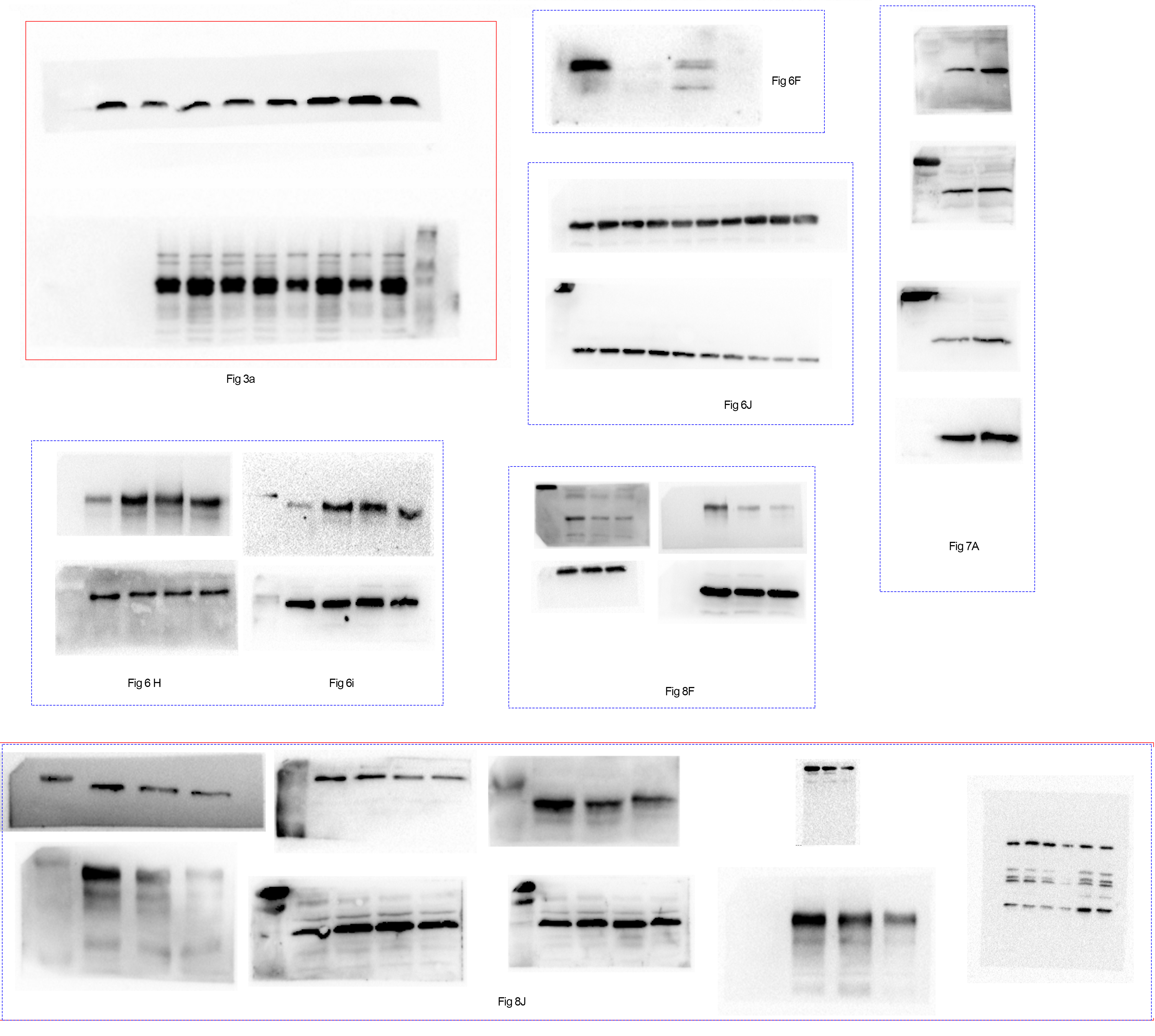

Supplement: Supplementary file 3 — Original WB images [file 41419_2024_6744_MOESM3_ESM.tif]
